# The Use of Polysaccharide AOP30 from the Rhizome of *Alpinia officinarum* Hance to Alleviate Lipopolysaccharide-Induced Intestinal Epithelial Barrier Dysfunction and Inflammation via the TLR4/NfκB Signaling Pathway in Caco-2 Cell Monolayers

**DOI:** 10.3390/nu16132151

**Published:** 2024-07-05

**Authors:** Xuejing Jia, Yun Huang, Guanghuo Liu, Zipeng Li, Qiwei Tan, Saiyi Zhong

**Affiliations:** Guangdong Provincial Key Laboratory of Aquatic Products Processing and Safety, Guangdong Provincial Science and Technology Innovation Center for Subtropical Fruit and Vegetable Processing, Guangdong Provincial Engineering Technology Research Center of Seafood, College of Food Science and Technology, Guangdong Ocean University, Zhanjiang 524088, China; jiaxj@gdou.edu.cn (X.J.); woej@stu.gdou.edu.cn (Y.H.); liuguanghuo@stu.gdou.edu.cn (G.L.); 202111221317@stu.gdou.edu.cn (Z.L.); tanqiwei@stu.gdou.edu.cn (Q.T.)

**Keywords:** *Alpinia officinarum* Hance, intestinal epithelial barrier, tight junction protein, TEER value

## Abstract

*Alpinia officinarum* Hance is rich in carbohydrates and is flavored by natives. The polysaccharide fraction 30 is purified from the rhizome of *A. officinarum* Hance (AOP30) and shows excellent immunoregulatory ability when administered to regulate immunity. However, the effect of AOP30 on the intestinal epithelial barrier is not well understood. Therefore, the aim of this study is to investigate the protective effect of AOP30 on the intestinal epithelial barrier using a lipopolysaccharide (LPS)-induced intestinal epithelial barrier dysfunction model and further explore its underlying mechanisms. Cytotoxicity, transepithelial electrical resistance (TEER) values, and Fluorescein isothiocyanate (FITC)–dextran flux are measured. Simultaneously, the protein and mRNA levels of tight junction (TJ) proteins, including zonula occludens-1 (ZO-1), Occludin, and Claudin-1, are determined using Western blotting and reverse-transcription quantitative polymerase chain reaction methods, respectively. The results indicate that AOP30 restores the LPS-induced decrease in the TEER value and cell viability. Furthermore, it increases the mRNA and protein expression of ZO-1, Occludin, and Claudin-1. Notably, ZO-1 is the primary tight junction protein altered in response to LPS-induced intestinal epithelial dysfunction. Additionally, AOP30 downregulates the production of TNFα via the Toll-like receptor 4 (TLR4)/NF-κB signaling pathway. Collectively, the findings of this study indicate that AOP30 can be developed as a functional food ingredient or natural therapeutic agent for addressing intestinal epithelial barrier dysfunction. It sheds light on the role of AOP30 in improving intestinal epithelial function.

## 1. Introduction

The intestine is a key organ in the digestive system, which is where the digestive process and absorption of nutrients takes place. After food is broken down in the stomach by acids and enzymes, it moves into the small intestine, where further digestion occurs; the intestine is where the absorption of nutrients such as carbohydrates, proteins, fats, vitamins, and minerals occurs, which indicates that the intestinal tract participates in the digestive process and nutrient absorption [1].

In addition, intestinal epithelial cells form a physical barrier that separates the contents of the intestine from the underlying tissues. This barrier also prevents harmful substances such as bacteria, toxins, and undigested food particles from crossing into the bloodstream [2]. Moreover, the intestinal epithelial barrier, which is located on the inner surface of the intestine and is made up of intestinal epithelial cells, is selectively permeable, allowing nutrients to be absorbed while blocking the entry of harmful substances. Thus, the intestinal epithelial barrier is also involved in the process of nutrient absorption.

However, the tight junction (TJ) of the intestinal epithelial barrier is one of the critical means of intestinal nutrient absorption. In fact, the TJ is a specialized structure that forms a seal between adjacent epithelial cells of the intestinal epithelial barrier and regulates the passage of ions, water, and solutes through the paracellular pathway [3]. Furthermore, the TJ acts as a physical barrier between intestinal epithelial cells, preventing the passage of harmful substances, pathogens, and antigens from the gut lumen into the bloodstream. This selective permeability is essential for maintaining gut homeostasis. The TJ is regulated by the movement of a protein complex, which occurs due to interactions between TJ proteins and membrane lipids within the apical and basolateral domains [4]. Additionally, this complex also plays an important role in maintaining the TJ’s polarity. Generally, the TJ protein primarily comprises zonula occludes-1, Occludin, and Claudin-1 [5]. When the TJ is weakened or disrupted, the integrity of the intestinal epithelial barrier becomes compromised; furthermore, this situation can lead to the increased permeability of the barrier, allowing harmful substances to pass through the epithelium and enter the bloodstream. Consequently, efforts to resolve intestinal barrier dysfunction have two challenges to address: (1) it increases intestinal permeability, which means that the organ can no longer prevent harmful substances like undigested food and toxins from entering the bloodstream; (2) it triggers inflammation in the gut. Therefore, it is urgent to develop a nontoxic agent to address these issues.

Encouragingly, polysaccharides offer hope of restoring intestinal epithelial barrier function.

Polysaccharides, derived from edible plants, are nontoxic biomacromolecules composed of a large number of monosaccharides, linked together through glycosidic linkages. These polysaccharides possess versatile biological activities, such as increasing the thymus and spleen indices of cyclophosphamide-induced immunosuppressed mice, downregulating the activity of α-glucosidase to reduce postprandial blood glucose levels, and inhibiting the proliferation of A549 lung cancer cells and HepG2 liver cancer cells [6,7,8]. Nevertheless, the issue of how to simulate intestinal epithelial barrier dysfunction is a problem that needs to be seriously considered.

Caco-2 cells are human cloned colon adenocarcinoma cells, which can be differentiated into intestinal epithelial cells and further formed into a monolayer. This layer possesses a similar structure and function to the intestinal epithelial barrier; hence, it is widely used to mimic the intestinal epithelial barrier, and extensive studies demonstrate this point. Luteolin mitigated brominated diphenyl ethers-209-induced barrier dysfunction in the Caco-2 cell monolayer [9]. Additionally, nanoparticles derived from porcine bone soup attenuated 1,1-diphenyl-2-picrylhydrazyl-radical-induced oxidative stress in a Caco-2 cell monolayer model [10]. These results indicate that a Caco-2-cell-formed monolayer is commonly utilized as an intestinal epithelial barrier model; moreover, LPS is a key component of the outer wall of Gram-negative bacteria. Moreover, taxifolin ameliorated a decrease in the TEER value in an LPS-induced caco-2 cell monolayer model [11]. Hence, LPS is a good actor for inducing dysfunction in the intestinal epithelial barrier when investigating the function of the epithelium.

*Alpinia officinarum* Hance is a herbaceous perennial agricultural product which was commonly regarded as a food spice that was added to porridge or soup in the south of China in order to drink them. Furthermore, this herb is made up of 20.25% carbohydrates [12]. Thus, a polysaccharide fraction of 30 was obtained from the rhizome of *A. officinarum* Hance (AOP30). Its molecular weight was 11.07 kDa and this polymer was composed of 89.88% glucose; importantly, AOP30 was a negatively charged macromolecule that displayed strong antioxidant activity against free radicals [13]. To date, there has been a lack of knowledge about the effects of AOP30 on the intestinal epithelial barrier. Thus, this study aims to provide insights into the role of AOP30 as bioactive constituents of food in alleviating intestinal barrier dysfunction and offer new directions as an ingredient for functional foods. Therefore, the aim of this study was to (i) determine its effect on the integrity of the intestinal epithelial barrier and (ii) identify the underlying mechanisms of AOP30 in protecting Caco-2 cells monolayers from LPS-induced barrier dysfunction based on molecular epithelial cell dysfunction.

## 2. Materials and Methods

### 2.1. Chemicals and Reagents

Lipopolysaccharide (LPS, *Escherichia coli* O55:B5, L5418-2ML) was obtained from Sigma-Aldrich Chemical Co., Ltd., (Shanghai, China). Dulbecco’s Modified Eagle Medium, fetal bovine serum (FBS), and a CCK−8 assay kit were obtained from zeta-life Invitrogen Co. (Guangzhou, China). A BCA protein assay kit (P0010) was purchased from the Beyotime Institute of Biotechnology (Shanghai, China). Hanks’ Balanced Salt Solution (HBSS) and nonessential amino acids were ordered from Guangzhou Solarbio Biotech Co., Ltd. (Guangzhou, China). Trypsin–EDTA solution was purchased from gibco Invitrogen Co. (Paisley, UK). Fluorescein isothiocyanate (FITC)–dextran (MW, 4000, GC18871) was bought from GLPBio Technology (Shanghai, China). Primary antibodies, including ZO-1 (21773-1-AP), Claudin-1 (13050-1-AP), Occludin (13409-1-AP), TLR4 (10494-1-AP), GAPDH (10494-1-AP), and phospho-NF-κB p65 (82335-1-RR), were supplied from proteintech Co., Ltd. (Wuhan, China). HRP-linked anti-rabbit IgG (7074) was purchased from cell signaling technology (Danvers, MA, USA). A TNFα enzyme-linked immuno-sorbent assay (ELISA) kit (MM-0122H2) was purchased from Meimian Industrial Co., Ltd., (Jiangsu, China). RNA isolation, reverse transcription, and qPCR kits were obtained from Vazyme Biotech Co., Ltd., (Nanjing, China). Superkine ECL solution was purchased from Abbkine scientific Co., Ltd., (Wuhan, China). The rhizome of *A. officinarum* Hance was collected from Longtang town (Guangdong, China) and AOP30 was prepared as described in the previous literature.

### 2.2. Cell Culture

Caco-2 cells (iCell-h032) were purchased from iCell Bioscience Inc. (Shanghai, China) and cultured at 37 °C and 5% CO_2_ atmosphere in a DMEM high-glucose medium supplemented with 10% FBS, 1% nonessential amino acid solution, and 1% penicillin−streptomycin liquid.

### 2.3. Cell Viability

Caco-2 cells were seeded in a 96-well plate at a density of 1 × 10^5^ cells/well and cultured overnight; then, cells were treated with different concentrations of AOP30 (100, 200, 400, and 800 μg/mL) for 24 h. Simultaneously, cells were exposed with 1 μg/mL of LPS. After that, the cells were incubated with 10 μL of CCK-8 solution for 1 h at 37 °C; lastly, the absorbance of each well was recorded at 450 nm by a Varioskan Flash (Thermo Scientific, Waltham, MA, USA). All the experiments were repeated three times.

### 2.4. Determination of Transepithelial Electrical Resistance

Transepithelial electrical resistance (TEER) was measured using a previously employed method [14] with minor modifications. In brief, 5000 caco-2 cells were seeded in a trans-well culture chamber (0.33 cm^2^, 3 μm, LAB select, Beijing, China). Its medium was changed every two days in the first week; then, it was replaced every day. TEER values were determined by an epithelium volt–ohm meter (RE1600, Beijing Jinhong Tai Technology Co., Ltd., Beijing, China) and the obtained values were calculated using the following formula: TEER = (Rt − Ri) × A. In this Ri, Rt, and A refer to the intrinsic resistance of a medium without cells, the resistance of a medium treated with targeted concentrations of AOP30, in Ω, and the surface area of the monolayer, in cm^2^. All these cells were cultured for 21 days until they formed a fully fused cell monolayer, where their resistance values were above 500 Ω. LPS was used to mimic epithelial barrier loss in vitro. The transepithelial electrical resistance (TEER) of Caco-2-plated filters was recorded by an epithelial volt–ohm meter after 24 h.

### 2.5. Determination of FITC–Dextran Paracellular Flux Analysis

The alteration of paracellular flux through Cacao-2 cell monolayers was measured by determining the apical-to-basolateral clearance of FITC–dextran. Caco-2 cells were treated as described to perform TEER determination. After the treatments, the culture medium was replaced by a HBSS solution in both compartments; thereafter, FITC–dextran (4 kDa) was added to the upper compartment with a final concentration of 100 μmol/L. After 3 h of further culturing in 37 °C, 100 μL of the solution in the lower compartment was collected and diluted with 100 μL of 1 × HBSS. The fluorescence intensity was measured at λ_exc_ 495 nm and λ_em_ 525 nm by a Varioskan Flash (Thermo Scientific, Waltham, MA, USA). All the experiments were repeated three times. 

### 2.6. Reverse Transcription-Quantitative Polymerase Chain Reaction Analysis

Total RNA was isolated from cells by the freezol reagent (R711-01, Vazyme); then, it was reverse-transcribed into cDNA using HiScript III Q RT supermix in a qPCR kit (R323-01, Vazyme). Subsequently, the level of mRNA expressions was determined using a ChamQ universal SYBR qPCR master mix kit (Q711-02, Vazyme); after that, the relative mRNA expression of the proteins (ZO-1, Claudin-1, Occludin, and glyceraldehyde 3-phosphate dehydrogenase (GAPDH)) was calculated using the comparative cycle threshold (2^−ΔΔCt^) method. GAPDH was performed as an endogenous control. Their primers were synthesized from Sangon Biotech Co., Ltd., (Shanghai, China) and are shown in Table 1.

### 2.7. Western Blotting Analysis

To measure the effects of the regulatory mechanism of AOP30 on LPS-induced dysfunction, expressions of TJ protein were determined via Western blot analysis. Caco-2 cells were seeded in a T25 culture flask at a density of 1 × 10^6^ cells/flask and incubated with AOP30 (200, 400, and 800 μg/mL) and 1 μg/mL of LPS overnight; after the treatment, the supernatant was collected to determine the production of TNFα, while cells were collected and lysed with a RIPA lysis buffer (Beyotime, P0013B). The total protein content was quantified according to the instructions of the BCA protein assay kit (P0010, Beyotime) and separated using 10% SDS−PAGE after denaturation. Electrophoresed proteins were transferred from gel to a 0.22 μm PVDF membrane; later, this was blocked with blocking solution (P0023B, Beyotime) for 1 h at room temperature. Primary antibodies acting against Occludin (13409-1-AP), Claudin-1(13050-1-AP), ZO-1(21773-1-AP), and GAPDH (10494-1-AP) were added to the membranes and incubated overnight at 4 °C. The membranes were washed three times to remove any unbound primary antibody and then incubated with appropriate horseradish peroxidase-conjugated secondary antibodies for 1 h at room temperature. Finally, the proteins were determined via reaction with a superkine ECL solution (ATWE11081, Abbkine) and exposure in a Tanon 5200 chemiluminescent image system after washing off unbound secondary antibodies. The intensity of immunoblot analysis was quantified using ImageJ software (v.1.50i, National Institutes of Health, Bethesda, MD, USA). 

### 2.8. ELISA Analysis

The level of TNFα in each treatment’s supernatant, described above, was determined according to the manufacturer’s instructions. Absorbance was measured at 450 nm using a Varioskan Flash (Thermo Fisher Scientific, Waltham, MA, USA). All the experiments were repeated three times.

### 2.9. Statistical Analysis

Each experiment was performed three times. Data are shown as mean ± standard deviation (SD), statistical analysis was performed using SPSS statistics 27.0 software, statistically significant differences among groups were analyzed by one-way analysis of variance (ANOVA), and a *p* < 0.05 was considered statistically significant.

## 3. Results

### 3.1. Cytotoxicity of AOP30 on Caco-2 Cells

**Figure 1:**
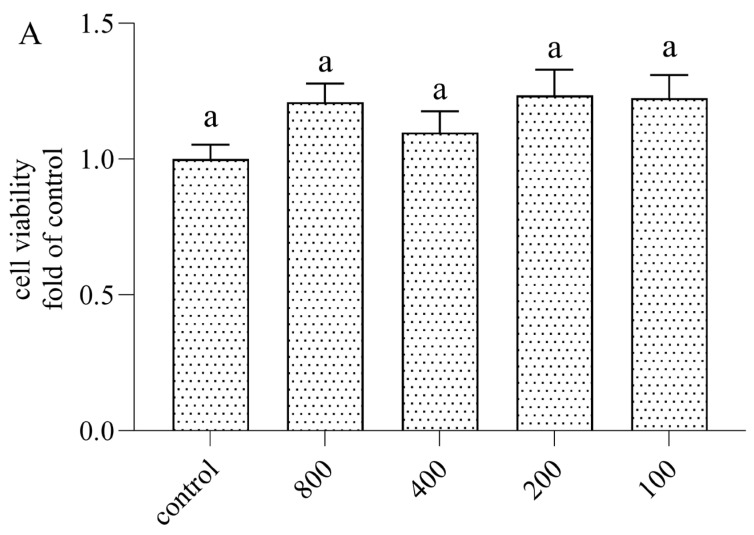

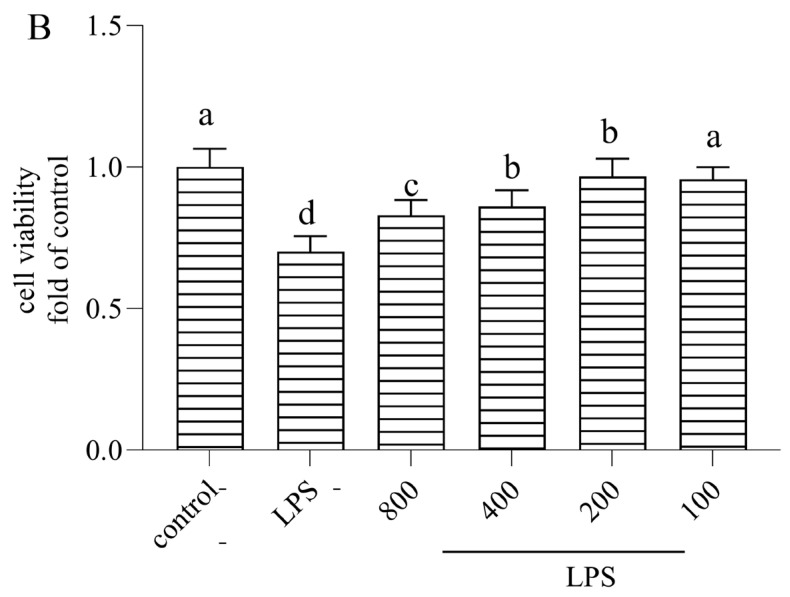
Effect of different concentrations of AOP30 (200, 400, and 800 μg/mL,), incubated alone (**A**) and co-incubated (**B**) with 1 μg/mL of LPS for 24 h, on cell viability in Caco-2 cells. Data are presented as mean ± SD. Bars with different superscript letters indicate significantly differences among the groups (*p* <  0.05).

The principle of cell-counting Kit-8 (CCK-8) was demonstrated by the dehydrogenation of live cells to a water-soluble orange-yellow formazan, which can be detected at 450 nm using a microplate reader. Furthermore, there was a positive correlation between the concentration of CCK-8 and the number of live cells. Therefore, this method showed the potential ability to evaluate a large number of cells at one time with high sensitivity. Thus, this high-throughput method was used for live cell detection [15]. To determine the cytotoxic potential of AOP30, the cell viability of Caco-2 cells was treated with different doses of AOP30 (100, 200, 400, and 800 μg/mL) alone and/or co-incubated with 1 μg/mL of LPS for 24 h. This was examined by the CCK-8 assay kit and the result is shown in Figure 1. There was no significant cell toxicity regarding the different concentrations of AOP30, ranging from 100 to 800 μg/mL (Figure 1A), or the incubation of AOP30 and LPS. Interestingly, AOP30 slightly promotes cell proliferation (Figure 1B). nevertheless, there was no difference between 100 μg/mL AOP30 and LPS groups. Thus, this result suggested that being co-cultured with different concentrations (100, 200, 400, and 800 μg/mL) of AOP30 might have a protective effect on LPS-induced intestinal dysfunction models.

### 3.2. AOP30 Attenuates LPS-Induced Decrease in TEER Value in Caco-2 Cell Monolayer

**Figure 2:**
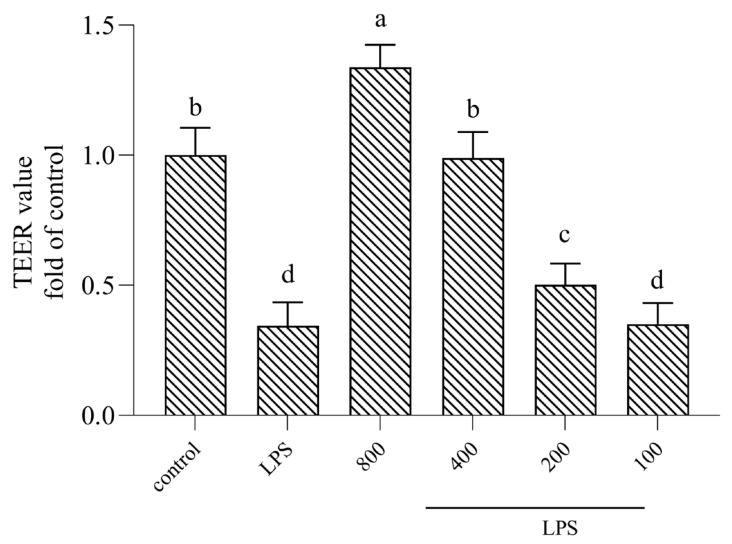
Effect of different concentrations of AOP30 (200, 400, and 800 μg/mL) co-incubated with 1 μg/mL of LPS for 24 h on TEER value in Caco-2 cells. Data are presented as mean ± SD bars. with different superscript letters indicating significantly differences among the groups (*p* < 0.05).

TEER played a crucial role in maintaining the homeostasis of the gut by selectively allowing the absorption of nutrients and preventing the entry of harmful substances, such as toxins, pathogens, and antigens. Additionally, the tight junctions between epithelial cells formed a physical barrier that regulated the paracellular transportation of molecules across the intestinal epithelium. Finally, TEER measurement involved applying a small electrical current across the epithelial monolayer and measuring the resistance to the flow of the current. A higher TEER value indicated a tighter barrier with reduced permeability, while a lower TEER value suggested compromised barrier function. Therefore, TEER measurements are commonly used in research studies to evaluate the effects of various factors on the intestinal epithelial barrier. Changes in TEER values can provide insights into the integrity of a barrier and its response to different stimuli. Overall, the TEER value was an important parameter for assessing the function and integrity of the intestinal epithelial barrier, which was essential for maintaining gut health and preventing the entry of harmful substances into the body. The TEER value was a quality indicator of the epithelial barrier that reflected paracellular permeability [16]. As shown in Figure 2, there was a dramatic decrease in the TEER value when cells were treated with LPS. However, when they were co-cultured with AOP30, this phenomenon was remarkably alleviated; in particular, the group treated with 800 μg/mL of AOP30 exhibited a stronger alleviating effect when compared to the other groups. surprisingly, no significant difference between 100 μg/mL AOP30 and LPS was observed (Figure 2). Combined with the above results regarding cytotoxicity, certain concentrations (200, 400, and 800 μg/mL) of AOP30 can be selected for further mechanism-related experimentation regarding this phenomenon.

### 3.3. AOP30 Alleviates LPS-Induced Alterations in FITC–Dextran Paracellular Transport in Caco-2 Cell Monolayer

**Figure 3:**
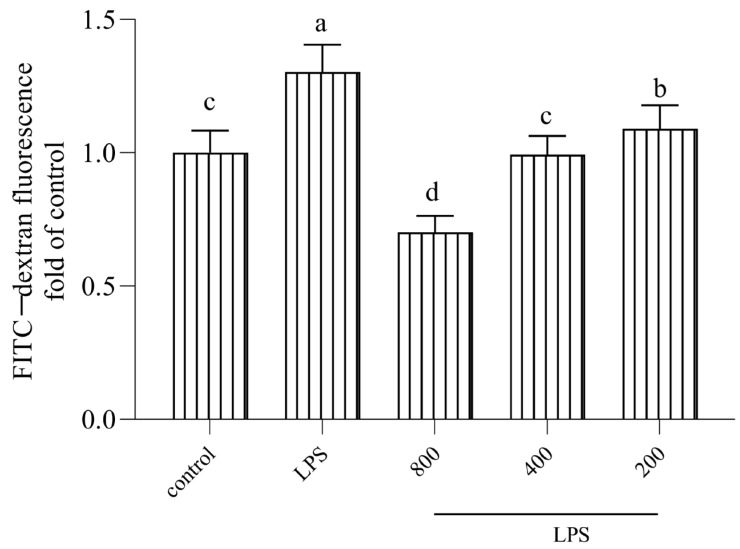
Effect of different concentrations of AOP30 (200, 400, and 800 μg/mL), co-incubated with 1 μg/mL of LPS for 24 h, on FITC–dextran paracellular transport; briefly, FITC–dextran (4 kD) was added into the upper chamber for 3.5 h after being replaced with HBSS solution. Thus, Caco-2 cell monolayer permeability was evaluated by measuring FITC–dextran paracellular transport. Data are presented as mean ± SD bars, with different superscript letters indicating significantly difference among the groups (*p* < 0.05).

To further confirm the alleviative effect of AOP30 on epithelial paracellular permeability, the effect of AOP30 on FITC–dextran transport in the Caco-2 monolayer was measured. As shown in Figure 3, 24 h of exposure to LPS resulted in a significant increase in the translocation of FITC–dextran from the upper to the lower chamber, indicating that the paracellular flux was altered, which was consistent with the results regarding TEER value. By contrast, treatment with different doses of AOP30 decreased paracellular permeability to FITC–dextran. Especially, 800 μg/mL AOP30 reduced the FITC–dextran fluorescence value by almost 50% compared to the LPS group (Figure 3). This result suggested that AOP30 protected the epithelial barrier against LPS-induced high paracellular permeability.

### 3.4. AOP30 Alleviates LPS-Induced Alterations in mRNA Expressions of Tight Junction Protein in Caco-2 Cells

**Figure 4:**
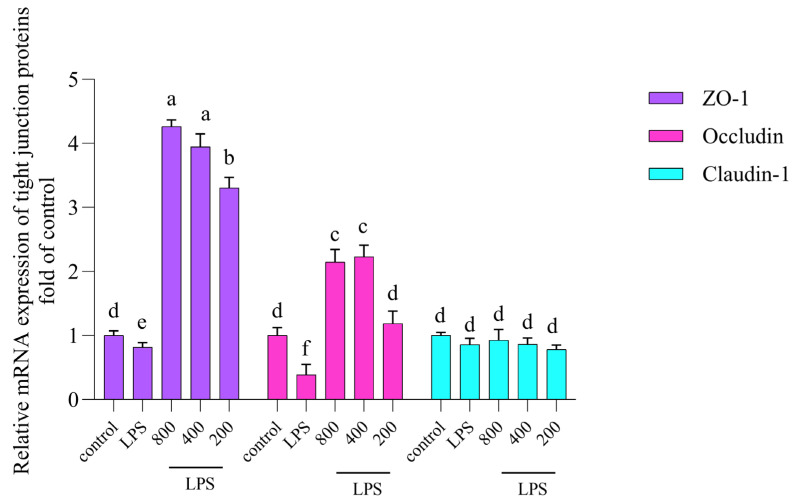
Effect of different concentrations of AOP30 (200, 400, and 800 μg/mL), co-incubated with 1 μg/mL of LPS for 24 h, on mRNA expression of ZO-1, Occludin, and Claudin-1 in Caco-2 cells. GAPDH is used as an internal reference, data are presented as mean ± SD, and bars with differing superscript letters indicate significant differences among the groups (*p* < 0.05).

Typically, mRNA levels provided information about the expression of specific genes in a cell. By measuring mRNA levels, we can assess the efficacy of a sample and determine whether genes are actively being transcribed. Thus, mRNA levels were closely correlated with protein levels [17]. Therefore, mRNA expressions of TJ proteins were determined. The results are shown in Figure 4. Cells were treated with LPS. The mRNA levels of ZO-1 and Occludin decreased, while no obvious decrease was found in Claudin-1. In fact, co-treatment with AOP30 increased the mRNA expression levels of ZO-1 and Occludin in LPS-treated Caco-2 cells. Particularly, 800 and 400 μg/mL of AOP30 produced the highest ZO-1 mRNA expression level (Figure 4), followed by Occludin. This result indicated that the TJ protein expression levels of ZO-1 and Occludin might be visibly altered. A similar study on Longan pulp polysaccharide alleviation of LPS-induced intestinal dysfunction supported this result [18]. Nonetheless, when co-cultured with different concentrations of AOP30, there no significant alteration in Claudin-1 levels was found, which suggested that the protein expression level of Claudin-1 does not respond to the stimulation of AOP30.

### 3.5. AOP30 Alleviates LPS-Induced Alterations of Tight Junction Protein Expressions in Caco-2 Cells

**Figure 5:**
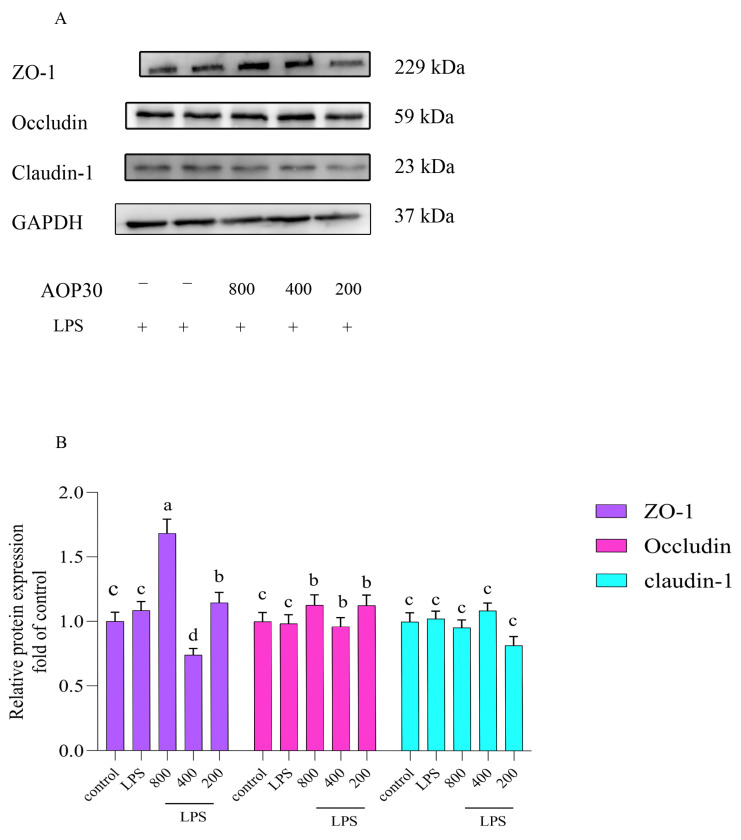
Effect of different concentrations of AOP30 (200, 400, and 800 μg/mL), co-incubated with 1 μg/mL of LPS for 24 h, on tight junction protein expression in Caco-2 cells. Western blotting analysis is conducted on the expression level of ZO-1, Occludin, and Claudin-1 (**A**,**B**), and GAPDH is used as an internal reference. Data are presented as mean ± SD; bars with differing superscript letters indicate significant differences among the groups (*p* < 0.05).

Proteins play essential roles in biological processes, protein alterations can occur, and changes in protein expression or modifications can serve as biomarkers for sample treatment. We ensured the quality and reliability of the experimental results, identified targets, and developed treatment strategies in order to confirm the reliability of the experimental results. Detecting alterations in proteins can provide insights into how they function. In this study, in an effort to explore the mechanism by which AOP30 promoted the TEER value of LPS-treated Caco-2 cells, TJ protein expressions were measured by Western blot analysis. The result is exhibited in Figure 5. LPS slightly decreased the expression of TJ proteins; conversely, AOP30 reversed this trend, which visually enhanced the expression of TJ proteins. ZO-1 showed higher expression than that of Occludin and Claudin-1. This might have been due to LPS disrupting the synthesis or assembly of ZO-1. Particularly, 800 μg/mL of AOP30 showed higher expression of ZO-1 than other groups. A similar result can be observed in that *Plantago asiatica* L. polysaccharides improved nonylphenol, induced a decrease in TJ protein, and highly augmented the expression of ZO-1 [19]. Nevertheless, there was no significantly alteration for Occludin. This might be owing to their differential mRNA expression (Figure 4), interestingly. The mRNA and protein expression of Occludin and Claudin-1 were consistent, which might be due to their posttranslational modifications. In summary, all these results revealed that AOP30 stimulated the preferential expression of ZO-1 in LPS-induced intestinal barrier dysfunction models. Intestinal epithelium served a defensive function by preventing luminal toxicants and infiltrating molecules [20]. When this barrier became dysfunctional, intestinal permeability increased, allowing unrecognized foreign substances to pass through and harm internal organs. Therefore, intestinal epithelial barrier permeability was a crucial factor for the intestinal barrier. Moreover, TJ acted as a physical barrier between adjacent epithelial cells, limiting the movement of molecules and ions [3], which perform consistent functions related to the intestinal epithelial barrier. Thus, TJ is necessary for intestinal epithelial cell barriers to absorb nutrients. Furthermore, it should be noted that this selective permeability was determined by the functioning of TJ. However, TJ function was controlled by the movement of the protein complex, which was the result of interaction between TJ proteins and membrane lipids within the apical and the basolateral domains [4]. In addition, this complex played a crucial role in TJ structure and function, helping to maintain epithelial polarity. Generally, TJ proteins comprise ZO-1, Occludin, and Claudin-1 [5]. As a matter of fact, ZO-1 was a cytosolic peripheral membrane protein with a specialized domain for protein interactions, which allowed it to bind with claudin-1 and Occludin [21]. Therefore, ZO-1 could be considered as a scaffold that organized proteins and formed a complex. In contrast, Occludin spanned the membrane and formed two extracellular loops of approximately the same size [22]. Thus, it should be regarded as a regulator involved in epithelial barrier dysfunction [23]. Nevertheless, Claudin-1 included two inconsistent sizes of extracellular loops that displayed some charged residues and had a function of sealing TJ [24]. Both Occludin and Claudin-1 were complementary molecules that could adhesively interact and were co-polymerized laterally [24]. Fortunately, it is reported that regulating the permeability may be attribute to the alteration of TJ protein expression [25]. Many studies confirmed this point. For example, *Lycium barbarum* polysaccharides prevented the loss of intestinal permeability in tumor-necrosis-factor-α-stimulated Caco-2 cells [26]; additionally, *Coix lachryma-jobi* L. polysaccharides reversed the decrease in TEER value in TNFα-challenged Caco-2 cells [27]. The *Scutellaria baicalensis* Georgi polysaccharide named SP2-1 increased the protein expression of ZO-1 and Occludin in the colon of dextransulfate sodium (DSS)-treated mice [28]; *Sargassum fusiforme* polysaccharides increased the mRNA and protein expression of ZO-1 and Occludin in the colon of mice with DSS-induced colitis [29]; pine pollen polysaccharides also promoted the expression level of ZO-1, Occludin, and Claudin-1 of colonic tissue in mice with DSS-induced ulcerative colitis [30]. Polysaccharides from the ink of *Ommastrephes bartrami* enhanced the protein expression level of Occludin and ZO-1 to protect the TJ function of cyclophosphamide-induced epithelial cell injury [31]. In addition, *Hericium erinaceus* polysaccharides augmented the expression level of occludin and ZO-1 to increase intestinal permeability [32]. Furthermore, *Lycium barbarum* polysaccharides prevented the release of pro-inflammatory cytokines and improved the protein expression levels of Claudin-1 and Occludin [26]. *Poria cocos* polysaccharides strengthened intestinal physical barrier by enhancing the protein expression level of ZO-1 and Occludin in the small intestine of mice [33]; hence, TJ exhibited a dynamic structure that presented evident permeability, i.e., TJ protein can regulate the permeability of intestinal epithelial barriers. TJ proteins and membrane lipids are dynamically interplayed to control TJ function [4]. In this study, the similar results we obtained agree with the above reports: different concentrations of AOP30 significantly decreased the cytotoxicity caused by 1 μg/mL of LPS in Caco-2 cells (Figure 1), indicating that LPS impairs Caco-2 cells. Moreover, the Caco-2 cell monolayer formed is disturbed by LPS. Apparently, the TEER value of the monolayer is decreased (Figure 2), as is the high translocation of FITC–dextran (Figure 3). Surprisingly, this disadvantageous situation is improved by treatment with AOP30. The permeability is improved by AOP30 treatment, which facilitates the protein expression of ZO-1, Occludin, and Claudin-1 (Figure 5). Notably, the function of the intestinal epithelial barrier depends on a healthy intestinal barrier with optimal permeability, which is helpful for absorbing nutrients. All these findings confirmed that AOP30 can improve the defensive and absorptive function of the intestinal epithelium.

### 3.6. AOP30 Downregulates the Production of TNFα via TLR4/NFκB Signaling Pathway

**Figure 6:**
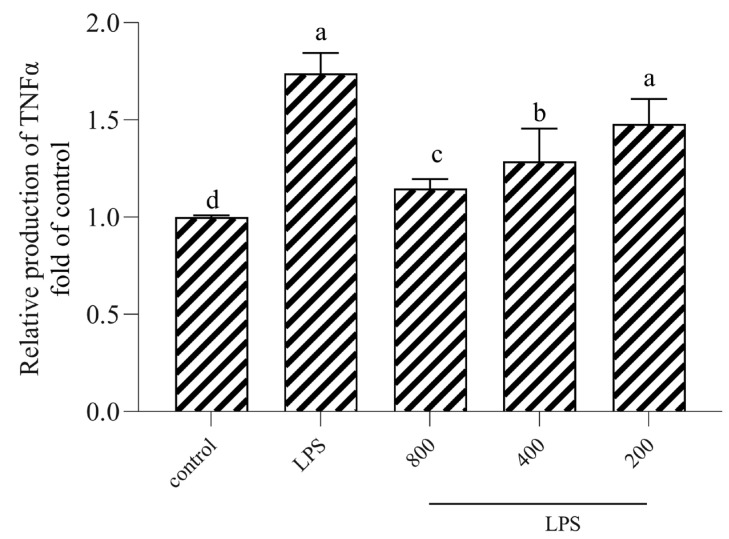
Effect of different concentrations of AOP30 (200, 400, and 800 μg/m) co-incubated with 1 μg/mL of LPS for 24 h on the secretion of TNFα in Caco-2 cells, data are presented as mean ± SD, and bars with differing superscript letters indicate significant differences among the groups (*p* < 0.05).

**Figure 7:**
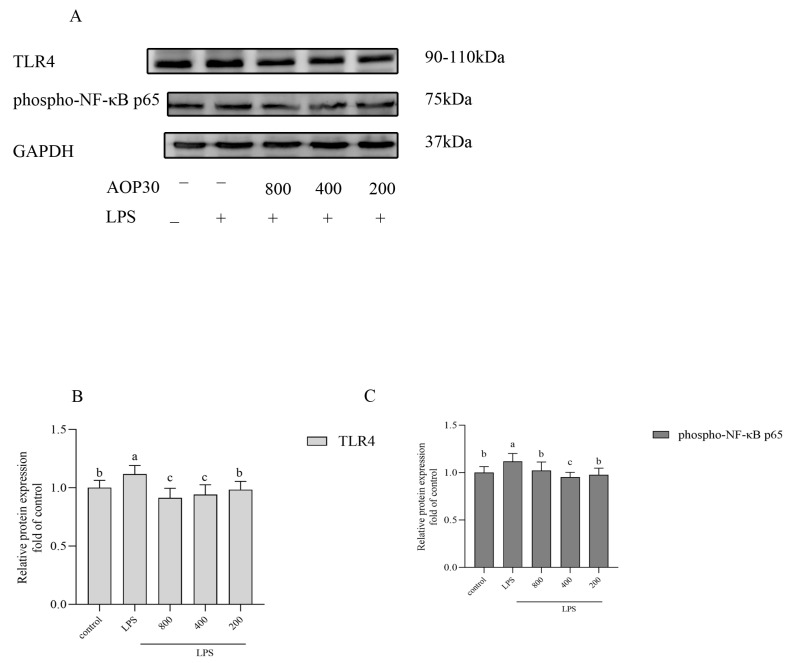
The effect of different concentrations of AOP30 (200, 400, and 800 μg/mL co-incubated with 1 μg/mL of LPS for 24 h on TLR4/NF-κB signaling pathway in Caco-2 cells. Western blotting analysis is conducted on the expression level of TLR4 (**A**,**B**), and phospho-NF-κB p65 (**A**,**C**). GAPDH is used as an internal reference, data are presented as mean ± SD, and bars with differing superscript letters indicate significant differences among the groups (*p* < 0.05).

The effect of AOP30 on the production of TNFα in the above supernatant was measured by the ELISA method, as shown in Figure 6. Treatment with high doses (800 and 400 μg/mL) of AOP30 significantly inhibited the release of TNF-α, while no difference between the low doses (200 μg/mL) of AOP30 and the LPS group was observed. This suggests that AOP30 may inhibit the production of TNF-α. Additionally, the NF-κB pathway is a critical regulator of inflammation [34]. Thus, we assume that AOP30 activates this pathway and leads to the secretion of TNF-α during inflammation. Therefore, the protein expression of TLR4 and phospho-NF-κB p65 were measured. As shown in Figure 7, inevitably, LPS increased the protein expression of TLR4 (Figure 7A,B) and phospho-NF-κB p65 (Figure 7A,C), whereas treatment with AOP30 decreased their expression levels, showing that AOP30 inhibited this signaling pathway following the LPS-based induction of dysfunction in Caco-2 cells. This suggests that AOP30 alleviates LPS-induced inflammation in Caco-2 cells.

## 4. Discussion and Conclusions

This study investigated the effect of AOP30 on LPS-induced intestinal epithelial barrier dysfunction. The results demonstrate that co-culturing with AOP30 can attenuate LPS-induced cytotoxicity, the transduction of FITC–dextran translocation and the decrease in TEER value, indicating that AOP30 promotes the intestinal permeability of Caco-2 monolayers. This result may be attributed to the increase in TJ protein expression. In particular, ZO-1 shows a promising target of AOP30 in terms of attenuating the dysfunction of the intestinal epithelial barrier. Moreover, AOP30 inhibits the production of TNFα via the TLR4/NF-κB signaling pathway. It is gratifying that AOP30 can improve intestinal epithelial barrier dysfunction, which may be due to the fact that AOP30 regulates the intestinal epithelial integrity and downregulates inflammatory level. Conclusively, AOP30 can be a promising agent for treating intestinal epithelial barrier dysfunction.

ZO-1 is important for maintaining intestinal barrier integrity [35]. Although the function of ZO-1 is unclear, a few studies conducted using in vivo and in vitro models demonstrated the important roles of ZO-1 in the TJ structure and permeability of the intestinal epithelia [3,36]. Unlike claudins, which mainly affect the flux of smaller-sized molecules and ions through a fixed pore, ZO-1 plays an important role in the flux of large macromolecules, such as inulin and dextran, through the paracellular barrier [37]. AOP30 blocks the inhibition of the protein expression of ZO-1 via LPS stimulus in Caco-2 cells in the present study (Figure 5 and Figure 7). Presumably, the anti-inflammatory effect ofAOP30 is partially due to the enhancement of ZO-1 expression, which is crucial to the selective flux of macromolecules, including bacterial antigens. Lastly, cumulative evidence supports the notion that this well-known treatment indeed has beneficial effects on gut health by increasing nutrient absorption, decreasing intestinal inflammation, and improving intestinal barrier function [38]. AOP30 significantly attenuated the reduction in phosphorylation of NF-κB p65 in the LPS-stimulated Caco-2 cell monolayer. In summary, AOP30 significantly prevented a reduction in the protein expression of ZO-1, attenuating the inhibition of the phosphorylation of NF-κB p65 compared with the LPS-treated group in Caco-2 cells, suggesting that intestinal integrity-protective effects of AOP30 are partially regulated by ZO-1 and NF-κB p65 activation.

In conclusion, AOP30 appeared to have potential intestinal barrier-promoting effects by reducing intestinal inflammation and promoting gut barrier function by partially regulating the tight junction-related proteins, ZO-1 and NF-κB p65, in human intestinal epithelial cells. A major limitation of our study is that the regulation of intestinal barrier function cannot be elucidated using an in vitro model alone due to the complexity of the intestinal barrier. In addition, further study is warranted to clarify the more precise mechanism of AOP30 by evaluating a diverse range of inflammatory cytokines. Collectively, we believe that our findings support the potential benefits of AOP30, which is considered as a functional food ingredient for alleviating intestinal barrier dysfunction.

## Figures and Tables

**Table 1:** Sequence of primers used in the study.

Gene Name	Forward Primer	Reverse Primer
ZO-1	GAGGTAGAACGAGGCATCATCCC	CTCCAGAAGTCAGCACGGTCTC
Occludin	ACTTCGCCTGTGGATGACTTCAG	TTCTCTTTGACCTTCCTGCTCTTCC
Claudin-1	AGGTACGAATTTGGTCAGGCTCTC	GGGACAGGAACAGCAAAGTAGGG
GAPDH	CACCCACTCCTCCACCTTTGAC	GTCCACCACCCTGTTGCTGTAG

## Data Availability

The raw data supporting the conclusions of this article will be made available by the authors on request.
